# Bayesian estimation of the prevalence of antimicrobial resistance: a mathematical modelling study

**DOI:** 10.1093/jac/dkae230

**Published:** 2024-07-25

**Authors:** Alex Howard, Peter L Green, Anoop Velluva, Alessandro Gerada, David M Hughes, Charlotte Brookfield, William Hope, Iain Buchan

**Affiliations:** Department of Antimicrobial Pharmacodynamics and Therapeutics, Institute of Systems, Molecular and Integrative Biology, University of Liverpool, William Henry Duncan Building, 6 West Derby Street, Liverpool L7 8TX, UK; Department of Medical Microbiology, Liverpool University Hospitals NHS Foundation Trust, Mount Vernon Street, Liverpool L7 8YE, UK; Civic Health Innovation Labs, University of Liverpool, Liverpool Science Park, 131 Mount Pleasant, Liverpool L3 5TF, UK; Civic Health Innovation Labs, University of Liverpool, Liverpool Science Park, 131 Mount Pleasant, Liverpool L3 5TF, UK; Department of Mechanical and Aerospace Engineering, School of Engineering, University of Liverpool, The Quadrangle, Brownlow Hill, Liverpool L69 3GH, UK; Department of Antimicrobial Pharmacodynamics and Therapeutics, Institute of Systems, Molecular and Integrative Biology, University of Liverpool, William Henry Duncan Building, 6 West Derby Street, Liverpool L7 8TX, UK; Civic Health Innovation Labs, University of Liverpool, Liverpool Science Park, 131 Mount Pleasant, Liverpool L3 5TF, UK; Department of Antimicrobial Pharmacodynamics and Therapeutics, Institute of Systems, Molecular and Integrative Biology, University of Liverpool, William Henry Duncan Building, 6 West Derby Street, Liverpool L7 8TX, UK; Department of Medical Microbiology, Liverpool University Hospitals NHS Foundation Trust, Mount Vernon Street, Liverpool L7 8YE, UK; Civic Health Innovation Labs, University of Liverpool, Liverpool Science Park, 131 Mount Pleasant, Liverpool L3 5TF, UK; Department of Health Data Science, Institute of Population Health, University of Liverpool, Waterhouse Building Block B, Brownlow Street, Liverpool L69 3GF, UK; Department of Medical Microbiology, Liverpool University Hospitals NHS Foundation Trust, Mount Vernon Street, Liverpool L7 8YE, UK; Department of Antimicrobial Pharmacodynamics and Therapeutics, Institute of Systems, Molecular and Integrative Biology, University of Liverpool, William Henry Duncan Building, 6 West Derby Street, Liverpool L7 8TX, UK; Department of Medical Microbiology, Liverpool University Hospitals NHS Foundation Trust, Mount Vernon Street, Liverpool L7 8YE, UK; Civic Health Innovation Labs, University of Liverpool, Liverpool Science Park, 131 Mount Pleasant, Liverpool L3 5TF, UK; Civic Health Innovation Labs, University of Liverpool, Liverpool Science Park, 131 Mount Pleasant, Liverpool L3 5TF, UK; Department of Public Health, Policy & Systems, Institute of Population Health, University of Liverpool, Waterhouse Building Block B, Brownlow Street, Liverpool L69 3GF, UK

## Abstract

**Background:**

Estimates of the prevalence of antimicrobial resistance (AMR) underpin effective antimicrobial stewardship, infection prevention and control, and optimal deployment of antimicrobial agents. Typically, the prevalence of AMR is determined from real-world antimicrobial susceptibility data that are time delimited, sparse, and often biased, potentially resulting in harmful and wasteful decision-making. Frequentist methods are resource intensive because they rely on large datasets.

**Objectives:**

To determine whether a Bayesian approach could present a more reliable and more resource-efficient way to estimate population prevalence of AMR than traditional frequentist methods.

**Methods:**

Retrospectively collected, open-source, real-world pseudonymized healthcare data were used to develop a Bayesian approach for estimating the prevalence of AMR by combination with prior AMR information from a contextualized review of literature. Iterative random sampling and cross-validation were used to assess the predictive accuracy and potential resource efficiency of the Bayesian approach compared with a standard frequentist approach.

**Results:**

Bayesian estimation of AMR prevalence made fewer extreme estimation errors than a frequentist estimation approach [*n* = 74 (6.4%) versus *n* = 136 (11.8%)] and required fewer observed antimicrobial susceptibility results per pathogen on average [mean = 28.8 (SD = 22.1) versus mean = 34.4 (SD = 30.1)] to avoid any extreme estimation errors in 50 iterations of the cross-validation. The Bayesian approach was maximally effective and efficient for drug–pathogen combinations where the actual prevalence of resistance was not close to 0% or 100%.

**Conclusions:**

Bayesian estimation of the prevalence of AMR could provide a simple, resource-efficient approach to better inform population infection management where uncertainty about AMR prevalence is high.

## Introduction

The prevalence of antimicrobial resistance (AMR) is one of the most widely used metrics in population infection management. Antimicrobial formularies, antimicrobial stewardship, infection prevention and control, and deployment of new drugs are examples of population infection management strategies that hinge on understanding AMR prevalence.^1,2^ For example, the prevalence of nitrofurantoin resistance in clinical urine specimens may inform whether nitrofurantoin should be used as a first-line treatment for urinary tract infection and/or whether it should be part of routine antimicrobial susceptibility testing panels; the prevalence of resistance to a new antimicrobial agent may influence its deployment in a new population (e.g. neonates) and the prevalence of MRSA may influence whether to implement screening programmes.

Real-world clinical microbiology data may not represent the true prevalence of AMR in the population for whom a decision needs to be made, for two reasons. Firstly, many people who have never had microbiology specimens sent have different patterns of AMR risk factors (e.g. different exposure to healthcare, different types and intensity of antimicrobial treatment), resulting in selection bias—this bias may be further compounded by reflex laboratory testing procedures (e.g. susceptibility testing only being performed for some antimicrobial agents when there is resistance to all other agents tested); secondly, estimates for seldom-tested antimicrobial agents are vulnerable to extreme estimation errors due to small sample sizes (e.g. identifying a single case of AMR for a drug with an AMR prevalence of 1:1000 by sampling 50 specimens).^3^

**Figure 1.**
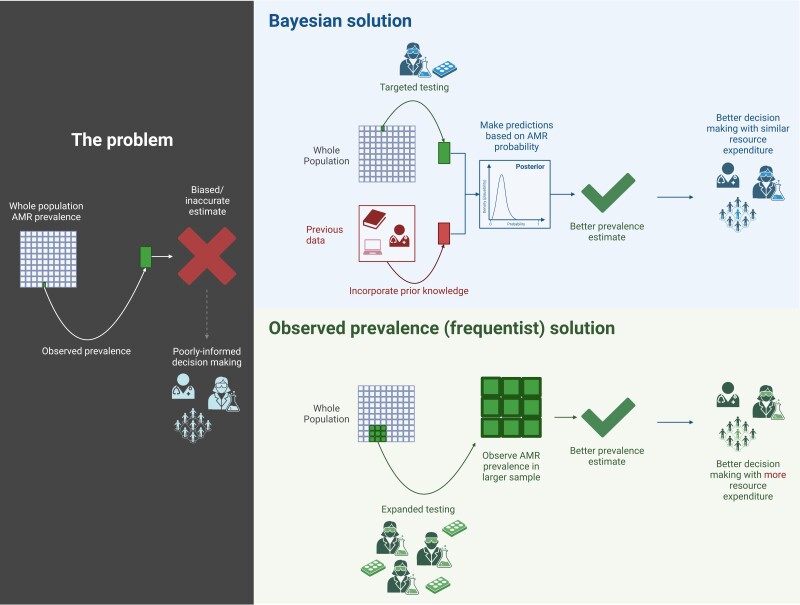
The problem with established practice in making population infection management decisions based on observed prevalence alone in biased subsets of data. The Bayesian solution is to incorporate prior data with targeted testing to improve predictions, while the frequentist solution necessitates expending additional resource to test a wider targeted population. This figure appears in colour in the online version of *JAC* and in black and white in the print version of *JAC*.

The standard, frequentist statistical approach to this problem is to test more specimens in the hope that the observed prevalence in a larger sample size begins to better represent the true population prevalence—this approach is resource intensive and often infeasible in busy, resource-constrained diagnostic laboratories.^4^ Bayesian inference is an alternative approach to frequentist statistics that allows the decision maker to combine prior information about the prevalence of AMR with that observed in the population sample (see Figure 1).^5,6^ This approach has two potential benefits: (i) the decision maker can utilize information about the wider, unsampled population thereby improving the representativeness of the estimate of AMR prevalence for the whole population; (ii) the information provided by prior belief addresses the need to test ever more specimens, potentially reducing resource expenditure in biomedical scientist time, consumables etc.

The aim of this study was to use a cross-validation approach^7^ on real-world antimicrobial susceptibility data to assess whether Bayesian Estimation of AMR prevalence (BEAR) could be a more reliable and more potentially resource-efficient way to estimate the prevalence of AMR in a new target population compared with standard frequentist methods.

## Methods

### Setting

The BEAR approach was developed and assessed using the open-source, pseudonymized real-world electronic healthcare record-derived dataset MIMIC (Medical Information Mart for Intensive Care)-IV version 2.2.^8–10^ The datasets used were those pertaining to hospitalwide inpatient and outpatient data for patients who were admitted to the ICU or the emergency department at Beth Israel Deaconess Medical Center (BIDMC) in Boston, MA, USA between 2008 and 2019.

### Participants and study size

Data for patients (denoted by pseudonymized subject identifiers) with specimens in the ‘microbiologyevents.csv’ MIMIC-IV dataset were analysed. Drug–pathogen combinations (listed in Table S1, available as Supplementary data at *JAC* Online) were chosen based on: (i) the number of available antimicrobial susceptibility results for them in the dataset, which reflected the ability to repeatedly select at least 20 results to inform the likelihood calculation; and (ii) expected phenotypes—drug–pathogen combinations affected by intrinsic resistance (e.g. *Proteus mirabilis* and nitrofurantoin) or expected susceptibility (e.g. tigecycline and *Staphylococcus aureus*) were not studied.

### Data sources/measurement and quantitative variables

Electronic health records were the source of all data in MIMIC-IV. The database literature does not detail what breakpoint interpretations were used to determine susceptible, ‘intermediate’ and resistant, but CLSI guidelines are the most likely interpretative criteria to have been used because they are recognized by the FDA in the USA. Organism identification processes used are also unknown and varied depending on organism and specimen type. For example, in urine cultures, all members of the *Klebsiella* genus were speciated but *Enterococcus* spp. were identified only to genus level.

### Development of the BEAR algorithm

**Figure 2.**
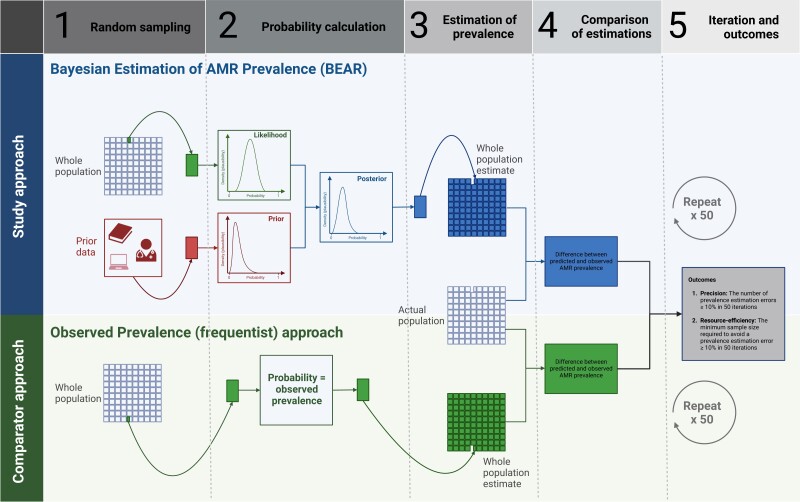
Parallel algorithm workflows for Bayesian and observed prevalence AMR estimation/validation. For each approach, 20 antimicrobial susceptibility results were sampled at random, then AMR prevalence was estimated in the remaining results. The differences between predicted and actual observed AMR prevalence were calculated for each approach, then the precision and resource efficiency of each approach were compared with descriptive statistics. This figure appears in colour in the online version of *JAC* and in black and white in the print version of *JAC*.

Algorithms were written and implemented using R version 4.3.2 (https://cran.r-project.org), which performed the following computation (also depicted in Figure 2) on retrospectively collected healthcare data: (i) authors’ prior belief in the population prevalence of AMR for each drug–pathogen combination was statistically expressed using a review of the literature^11–28^ contextualized by authors, summarized in Box S1 and Figure S1; (ii) the dataset was filtered for drug–pathogen combinations of interest where an antimicrobial susceptibility test result was available, then 20 susceptibility test results for each pathogen were sampled at random—these 20 results represent the specimen antimicrobial susceptibility results that would be available to the decision maker in real-world practice; (iii) BEAR calculated the probability distribution of the prevalence of AMR that was observed in the sampled 20 results (i.e. the probability of observing that prevalence of AMR conditional on all possible probabilities of AMR in any single case—otherwise known as the Bayesian likelihood); (iv) BEAR calculated the posterior probability of resistance from the prior and the observed data using Bayes’ theorem (see Box S2), then used this probability distribution to generate a posterior predictive distribution that estimated the prevalence in the unobserved results; (v) 49 further iterations of steps (ii) to (iv) were repeated and the differences between predicted and observed AMR prevalence (estimation error) were measured over these iterations; (vi) steps (ii) to (v) were then performed repeatedly with an ascending result sample size until no estimation errors  ≥10% were recorded in 50 iterations; (vii) the whole analysis was then repeated using a comparator frequentist approach that estimated AMR probability based purely on the observed AMR prevalence in the sampled 20 results; and (viii) the results of the BEAR and the observed prevalence approach cross-validations were compared using summary statistics and data visualization techniques.

To facilitate a simplified binomial model of probability estimation, ‘intermediate’ results (results that are neither susceptible nor resistant, with a variable definition and interpretation depending on regional susceptibility testing guidelines)^29,30^ were interpreted as susceptible in the main analysis. This approach was taken because selection bias is more likely to result in an overestimation of resistance (for example, urine cultures only being sent for patients who have not responded to treatment)—interpreting ‘intermediate’ as resistant risks further exacerbating this bias and blunting the effect of BEAR on real-world estimates. However, there are some clinical situations where a more clinically conservative estimate of resistance would be appropriate (for example, in informing critical care guidelines). A sensitivity analysis was therefore performed where ‘intermediate’ results were reclassified as resistant, the algorithms were run again, and the results were compared with the main analysis by box plot data visualization.

### Variables

The outcomes of interest were: (i) estimation precision—the number of extreme AMR prevalence estimation errors (≥10% difference between estimated and actual AMR prevalence) in 50 cross-validation iterations of BEAR and the observed prevalence approach using 20 observed antimicrobial susceptibility results; the number of very extreme errors (≥20%) was a secondary outcome; and (ii) potential resource efficiency—the minimum number of observed antimicrobial susceptibility results per drug–pathogen combination required to avoid any extreme errors in AMR prevalence in 50 cross-validation iterations of BEAR versus the observed prevalence approach.

### Statistical methods

BEAR calculated the posterior probability of AMR, based on Bayes’ theorem, and simulated results using the resultant beta-binomial posterior predictive distribution (a beta prior was chosen as a conjugate prior to provide a closed form expression for the posterior—this is described by Equations 1–4 in Box S2). AMR probability was calculated by the comparator observed prevalence approach using only the prevalence of AMR in the 20 sampled results. The non-sampled results in the dataset were then replaced with results simulated based on these probability calculations using R’s ‘rbetabinom’ function. The estimation error was defined as the difference between the prevalence of AMR in these simulated results versus the actual percentage prevalence of AMR in the results they replaced. The precision outcome was the difference in the number of prevalence estimation errors  ≥10% (extreme errors) between BEAR and the observed prevalence approach in 50 cross-validation iterations. The resource-efficiency outcome was the minimum observed sample size per drug–pathogen combination required to avoid any prevalence estimation errors >10% for BEAR and the observed prevalence approach in 50 cross-validation iterations. Tests of statistical significance were not suitable for the cross-validation approach used, because the power to reject the null hypothesis would be determined by the number of iterations, which could be arbitrarily increased or decreased as required.

## Results

### Participants

Figure S2 displays the flow chart for the analysed datasets. In the raw ‘microbiologyevents.csv’ dataset, 1 587 215 specimens pertaining to 181 226 patients were present. During data cleaning, 33 383 specimens were removed because they contained the following terms not recognized and parsed correctly by the R ‘AMR: Antimicrobial Resistance Data Analysis’ package version 2.1.1 (available at https://cran.r-project.org/web/packages/AMR/index.html): ‘cancelled’, ‘virus’, ‘simplex’, ‘parainfluenza’, ‘influenza A’, ‘influenza B’, ‘tick’, ‘AFB grown’, ‘Gram variable rods’, and ‘Hymenolepis’. A total of 89 643 specimens from 41 531 patients were determined to be eligible for cross-validation analysis based on the criteria described in the ‘Participants and study size’ section.

### Descriptive data

**Figure 3.**
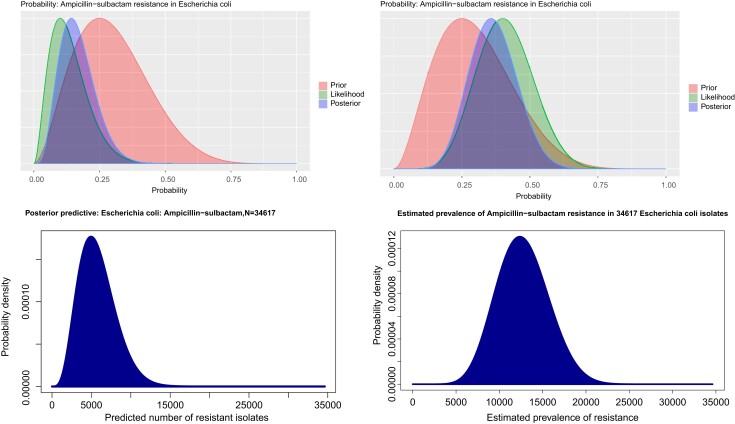
An example output of the BEAR approach for ampicillin/sulbactam resistance in *E. coli*, from two random samples of 20 specimens in the study dataset. The prior (red curve, left-hand graphs) reflects our belief that we think the prevalence of resistance is likely to be less than 50%, but not very rare, based on the prevalence of ampicillin/sulbactam resistance in inpatient and outpatient epidemiological studies in the USA—the mode of the prior is therefore around 0.25. The likelihood (green curve, left-hand graphs) reflects the prevalence of resistance that has been observed in the 20 sampled results—making an estimate based on the data alone would result in almost a 30% swing in the estimate depending on whether the top sample (observed prevalence ∼10%) or the bottom sample (observed prevalence ∼40%) had been used to make the estimate. Combining the prior and the likelihood results in the posterior (blue curve, left-hand graphs), which reflects our Bayesian estimate of AMR prevalence in the dataset—the anchoring effect of the prior prevents the posterior (the blue curve in the left-hand plots) from overfitting to each sample, significantly reducing the amount of swing in estimate between samples. The right-hand graphs show differences in the resulting predictions in the population context—the Bayesian approach limits the swing of estimates between samples to around 7000 for a population of 34 617, anchoring the estimate closer to the actual whole population prevalence of 8141. Using the observed data alone would have resulted in a swing of around 10 000, and a less accurate estimate in both cases.

The characteristics of the study population are displayed in Table 1. Figure 3 displays outputs of BEAR run on two random samples of 20 susceptibility results to estimate prevalence of ampicillin/sulbactam resistance in an unobserved population of 34 617 *Escherichia coli* isolates.

**Table 1.** Characteristics of the study population

Measure(s)	Summary statistic(s)
Age, years, median (IQR)	63 (47–76)
Gender female (F), *n* (%)^a^	24 955 (60.1)
**Specimen type, *n* (%)**	
Sterile specimens^b^	11 797 (13.2)
Inpatient specimens	34 432 (38.4)
**Organism grown, *n* (%)**	
*Citrobacter freundii*	1050 (1.2)
*Enterobacter cloacae*	2788 (3.1)
*Enterococcus* spp.	11 828 (13.1)
*E. coli*	34 651 (38.6)
*Klebsiella pneumoniae*	11 084 (12.4)
*Morganella morganii*	699 (0.7)
*P. mirabilis*	4492 (5.0)
*Pseudomonas aeruginosa*	8694 (9.7)
*Serratia marcescens*	1425 (1.6)
*S. aureus*	23 617 (26.3)
*Stenotrophomonas maltophilia*	1382 (1.5)
*Streptococcus* Group B	748 (0.8)
*Streptococcus pneumoniae*	643 (0.7)

^a^MIMIC-IV provides a column listed ‘gender’ with the binary elements ‘M’ and ‘F’. In the absence of further available information, we have not assumed whether this refers to gender or sex and have summarized the data as provided.

^b^Sterile specimens were defined as those from blood, tissue or fluid confirmed to be from a sterile compartment (e.g. CSF).

### Main results

**Figure 4.**
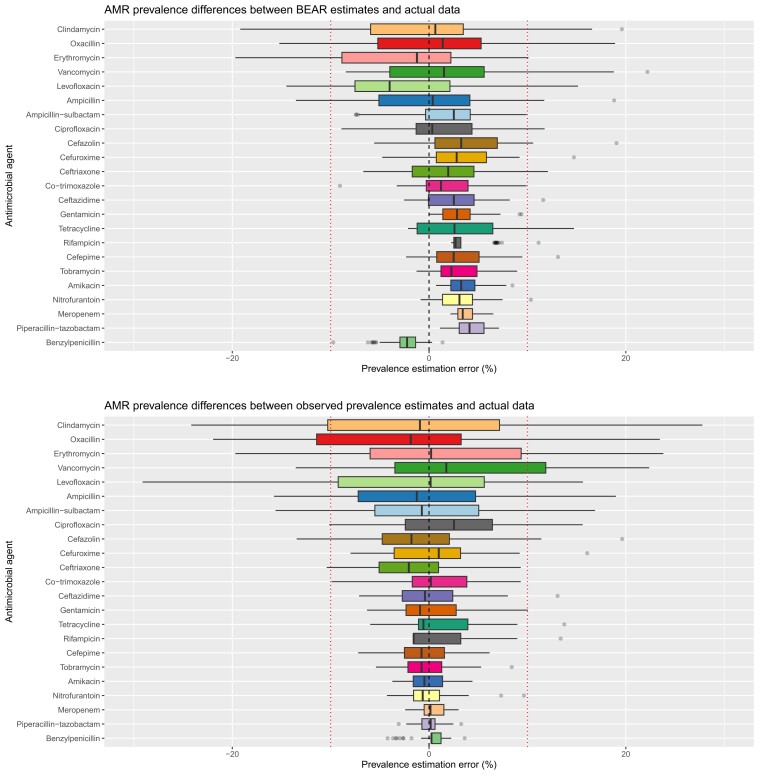
Box plot displaying distribution of AMR estimation errors incurred during cross-validation using BEAR and the frequentist observed prevalence approach. BEAR resulted in fewer errors ≥10% and fewer errors ≥20% than the observed prevalence approach. This figure appears in colour in the online version of *JAC* and in black and white in the print version of *JAC*.

Table 2 summarizes AMR prevalence estimation error for BEAR and the observed prevalence approach across the cross-validation (actual and estimated AMR prevalence used to compute errors for each method are summarized in Table S2). Each algorithm used 2160 observed results to simulate 919 057 deleted results per cross-validation iteration. Using BEAR resulted in fewer AMR prevalence estimation errors than the observed prevalence approach [*n* = 74 (6.4% of estimations) versus *n* = 136 (12.2% of estimations)] across the cross-validation. This is reflected by the distribution of estimation errors in 50 cross-validation iterations shown in Figure 4—the mean of estimates for BEAR were often further from the true population value than the observed prevalence approach, but there was a much smaller spread of estimate errors with BEAR than with the observed prevalence approach. BEAR made a single estimation error ≥20% for vancomycin, versus 14 errors ≥20% made by the observed prevalence approach [for oxacillin (*n* = 7), levofloxacin (*n* = 2), erythromycin (*n* = 1), clindamycin (*n* = 3) and vancomycin (*n* = 1)]. For drug–pathogen combinations where the actual prevalence of resistance was very low (approaching 0%) or very high (approaching 100%), the difference between BEAR and the observed prevalence approach appeared to narrow and BEAR tended to slightly overestimate prevalence, albeit within a 10% margin of error.

**Table 2.** Summary measures of estimation errors across all cross-validations for BEAR and the observed prevalence approach

		BEAR	BEAR	Observed prevalence	Observed prevalence
Antimicrobial agent	Number of results tested (and simulated)	Mean % AMR prevalence estimation error (SD)	Number of prevalence estimation errors ≥10%	Mean % AMR prevalence estimation error (SD)	Number of prevalence estimation errors ≥10%
Benzylpenicillin	40 (3204)	−2.6 (2.1)	0	0.2 (1.7)	0
Ampicillin	80 (50 396)	0.1 (6.6)	8	−0.3 (8.3)	13
Oxacillin	20 (23 577)	0.4 (7.7)	10	−0.4 (13.1)	21
Ampicillin/sulbactam	60 (49 035)	1.8 (4.2)	0	−0.2 (7.2)	8
Piperacillin/tazobactam	80 (52 747)	4.2 (1.5)	0	−0.1 (1.3)	0
Cefazolin	60 (46 695)	3.5 (4.7)	4	−1.3 (6.1)	6
Cefuroxime	60 (7036)	3.1 (4.1)	1	0.5 (5.2)	1
Ceftriaxone	60 (48 984)	1.9 (4.5)	4	−1.7 (4.9)	1
Ceftazidime	80 (57 708)	2.5 (3.2)	1	0 (4)	1
Cefepime	180 (63 430)	3 (3.2)	1	−0.5 (3.3)	0
Meropenem	180 (63 548)	3.6 (1)	0	0.2 (1.4)	0
Ciprofloxacin	180 (63 571)	1 (4.9)	3	2.2 (6)	6
Levofloxacin	40 (23 673)	−2 (7)	8	−0.6 (11.4)	16
Erythromycin	60 (23 128)	−2.6 (7.5)	10	0.9 (10.7)	17
Clindamycin	40 (21 085)	−0.7 (8.5)	13	−1 (11.2)	23
Tetracycline	40 (17 542)	3.3 (4.1)	5	0.6 (4.6)	1
Vancomycin	20 (11 801)	1.7 (6.9)	4	2.2 (9.8)	20
Rifampicin	20 (8629)	3.7 (2.1)	1	0.4 (3.5)	1
Gentamicin	200 (87 137)	3.2 (2.2)	0	0.1 (3.5)	1
Amikacin	120 (5205)	3.7 (2)	0	−0.1 (2.2)	0
Tobramycin	180 (63 554)	2.8 (2.3)	0	−0.3 (2.9)	0
Nitrofurantoin	140 (47 669)	3.1 (2.3)	1	0 (2.6)	0
Trimethoprim/sulfamethoxazole	220 (79 703)	1.7 (3.5)	0	0.7 (4.9)	0
Total	2160 (919 057)		74		136

**Figure 5.**
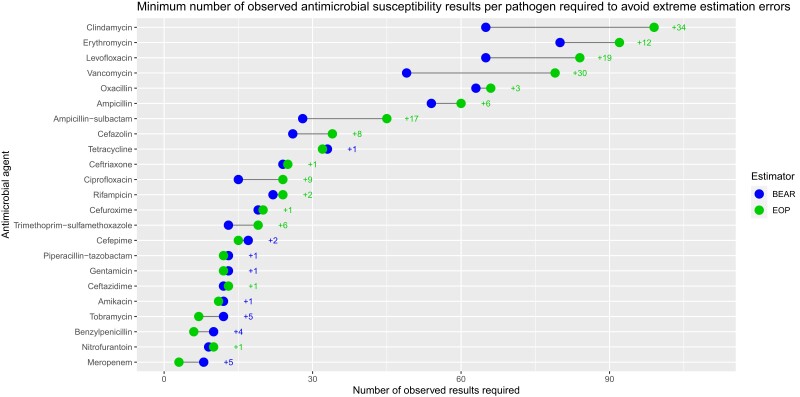
The minimum number of antimicrobial susceptibility results per drug–pathogen combination that were needed to avoid any extreme (≥10%) AMR prevalence estimation errors in 50 cross-validation iterations of BEAR and the estimation using observed prevalence (EOP) approach. This figure appears in colour in the online version of *JAC* and in black and white in the print version of *JAC*.

Table 3 and Figure 5 summarize the results of the laboratory resource efficiency analysis. BEAR required a smaller minimum number of antimicrobial susceptibility results per drug–pathogen combination to avoid any AMR prevalence estimation errors ≥10% (mean = 28.8, SD = 22.1 versus mean = 34.4, SD = 30.1). Similarly to the precision analysis, the resource benefit of BEAR over the observed prevalence approach generally appeared to narrow for drug–pathogen combinations where the actual prevalence of AMR was very low or very high.

**Table 3.** The number of antimicrobial susceptibility results per pathogen that were needed to avoid any extreme (≥10%) AMR prevalence estimation errors in 50 cross-validation iterations of BEAR and the observed prevalence approach

	BEAR	Observed prevalence
Antimicrobial agent	Minimum required number of observed results per pathogen	Minimum required number of observed results per pathogen
Benzylpenicillin	10	6
Ampicillin	54	60
Oxacillin	63	66
Ampicillin/sulbactam	28	45
Piperacillin/tazobactam	13	12
Cefazolin	26	34
Cefuroxime	19	20
Ceftriaxone	24	25
Ceftazidime	12	13
Cefepime	17	15
Meropenem	8	3
Ciprofloxacin	15	24
Levofloxacin	65	84
Erythromycin	80	92
Clindamycin	65	99
Tetracycline	33	32
Vancomycin	49	79
Rifampicin	22	24
Gentamicin	13	12
Amikacin	12	11
Tobramycin	12	7
Nitrofurantoin	9	10
Trimethoprim/sulfamethoxazole	13	19
Mean (SD)	28.8 (22.1)	34.4 (30.1)

### Other analyses

The results of the sensitivity analysis for ‘intermediate’ susceptibility results are summarized in Table S3 and Figure S3. Reclassification of ‘intermediate’ results to resistant did not substantially affect the results of the analysis—BEAR still resulted in fewer estimation errors ≥10% than the observed prevalence approach ([*n* = 75 (6.5% of estimations) versus *n* = 127 (11.0% of estimations)]. BEAR made a single estimation error ≥20% for erythromycin, versus five errors ≥20% made by the observed prevalence approach [ampicillin/sulbactam (*n* = 1), levofloxacin (*n* = 1), erythromycin (*n* = 1) and clindamycin (*n* = 2)].

## Discussion

It is less probable that the Bayesian approach (BEAR) will make extreme prevalence estimation errors than a frequentist approach in cases where there are few results available and there is a moderate risk of resistance. BEAR requires fewer datapoints to make timely and robust estimates of the prevalence of AMR because it adds contextualized prior information to the observed data—to achieve the same effect, the frequentist approach needs to progressively add more data from the population and test ever more specimens. The benefit of a prior is to anchor estimates in a believable range, preventing extreme estimates (caused by a phenomenon called overfitting) that could be excessively harmful or wasteful when used to make population infection management decisions (e.g. choosing a first-line agent for an antimicrobial formulary). Our study is, to our knowledge, the first time a simple Bayesian model of this kind has undergone implementation-focused investigation for AMR.

Given the prior distributions used in this study, the Bayesian approach provides less or no benefit over observed prevalence alone for drug–pathogen combinations where the actual prevalence of AMR is either very high or very low, because in those situations, observed prevalence alone is more likely to make accurate estimates by chance. For example, because meropenem resistance is rare, most sampling frames of 20 in the dataset will have no meropenem resistance in them, likely resulting in an estimate of zero with the observed prevalence approach—by chance, this is very close to the correct answer. It is for this reason that frequentist approaches require tests of statistical significance to quantify the probability that an observed phenomenon in a small sample happened by chance. Contrast this with ampicillin/sulbactam (see the example in Figure 3), where there is likely to be much more variation in observed prevalence between sampling frames of 20, so the probability of making an extreme error based on a small sample of observed data alone is higher—Bayesian approaches provide more benefit here because the prior provides much-needed additional information to this more variable and unpredictable scenario. BEAR may therefore have less utility in certain clinical situations—for example, in estimating the prevalence of carbapenemase-producing Enterobacterales in low-prevalence settings, or estimating the prevalence of phenoxymethylpenicillin resistance in *S. aureus*.

BEAR could be relevant in many different types of infection-related decision making—consider three scenarios that are commonly encountered in infection-related healthcare. Firstly, to determine whether ampicillin/sulbactam should be recommended as a first-line treatment for urinary tract infection in community healthcare settings, public health databases could be used to quantify prior belief about the context-specific prevalence of ampicillin/sulbactam resistance. BEAR uses this Bayesian prior with a limited number of local urine antimicrobial susceptibility results (28 specimens per pathogen was sufficient to avoid extreme estimation errors in this study) to obtain a rapid, resource-efficient and robust estimate of prevalence to guide whether ampicillin/sulbactam should be used for the population in question. This specific example highlights a further advantage of BEAR in that many patients do not have urine specimens sent—missing data scuppers estimates of prevalence, and this is especially difficult if standard frequentist approaches are used. BEAR circumvents this issue with the use of a prior that enables prior knowledge to be incorporated when there are missing data, as is often the case in infection management.

A further example relates to the deployment of a newly developed antimicrobial agent or an agent for use in a special population. In either case, there is a paucity of data to guide policy development and aid decision-making. Consider the use of a new agent for the treatment of neonatal sepsis. Information from a variety of sources (e.g. clinical, microbiological, pharmacological datasets) alongside available data from separate populations (e.g. adults) helps form a contextualized prior belief of the use of the new drug in neonates. BEAR is then used to combine this belief with focused testing that is designed to be representative of the new target population. This approach enables an informed decision of likely efficacy of the new drug across neonatal populations.

Finally, consider the national deployment of an agent with ability to address unmet medical needs via activity against MDR/XDR pathogens. Here, information contained in the non-clinical and clinical development programmes contributes to the prior, which effectively provides a summary of contemporaneous knowledge. A strategically designed sampling programme provides data likely to be representative of the true within-country prevalence of AMR. BEAR is then used to make estimates of the likely efficacy of the new agent without requiring an exhaustive number of datapoints.

Where ample, unbiased population data are available (e.g. in a well-designed large-scale study with active microbiological screening), a simple Bayesian approach like BEAR is unlikely to have significant benefit over a traditional frequentist technique—however, large-scale screening studies of this kind are slow and resource intensive. A further disadvantage of such datasets is that once collected they are immediately out of date. One of the characteristics of antimicrobial therapy and AMR is that the quality and extent of knowledge is highly dynamic and continuously changing. BEAR facilitates responsive and real-time clinical decision-making and design/implementation of dynamic public health policy. As new information becomes available, the prior can be updated, and the Bayesian estimates of AMR prevalence recalculated to assess whether new clinical and public health interventions are required. Such an approach is an example of the use of feedback loops that are essential for the development of modern resilient healthcare systems that make best use of data collected in routine healthcare and/or from surveillance programmes. Feedback loops of this kind could also be used to deploy the intervention across a range of geographical settings at scale, by using prior results to inform estimates in neighbouring and/or similar geographical areas. BEAR also has a role in AMR preparedness (consider similarities to the recent SARS-CoV-2 pandemic) by providing a new tool to address emergent public health threats that require rapid decision making and public health interventions.

With deployment in real-world settings comes the need for effective monitoring and quality control. Statistical techniques such as the cross-validation approach used in this study should be combined with the data visualization techniques provided by the algorithm (as shown in Figure 3), clinical outcomes (e.g. the frequency of WHO Access, Watch and Reserve agents in an antimicrobial formulary that has been based on BEAR estimates) and fidelity metrics (e.g. uptake of BEAR by infection specialists) to ensure BEAR remains safe, efficient and fit for purpose once deployed in clinical practice. It is also important to understand the subpopulations that available data come from, and the assumptions made when making estimates—for example, priors derived from susceptibility data in outpatient settings may translate poorly to inpatient settings where resistance prevalence is likely to be higher. In such situations, mathematical weighting could be applied to illustrate lower confidence in the priors, or targeted additional data collected to increase the weight of observed data in calculations.

Our new approach has several limitations. Firstly, the cross-validation method we used is likely to have overestimated whole-population precision and resource efficiency of the observed prevalence approach, because it validated against a selected population (i.e. patients who had microbiology tests sent) rather than the whole population. The population data used also only related to a specific geographical setting in adult patients—to better test the utility of BEAR in the example scenarios described above, the approach should be validated on data from different populations and settings. Secondly, how well the incorporation of prior belief mitigates selection bias depends how well that belief reflects reality—this could present a challenge in some settings where the availability of literature and/or data to inform priors may be lacking. Thirdly, the number of cross-validation iterations was limited to 50 for each drug–pathogen combination to limit the cumulative time taken for the algorithm to compile results. Fourthly, ≥10% estimation error was chosen as a potentially clinically significant extreme error, but the clinical significance of a ≥10% error varies from drug to drug, pathogen to pathogen, and cohort to cohort.

Despite these potential limitations, our results suggest that system leaders in antimicrobial stewardship, infection prevention and control, and drug development could use BEAR as a simple, reliable, resource-efficient way of estimating AMR prevalence when making population infection management decisions. The usefulness of policies developed in these domains (for example, antimicrobial formularies) could be strengthened by the ability to more accurately estimate AMR prevalence with existing testing resources using BEAR—this would have potential benefits both in terms of individual management of infection and helping to control the population spread of AMR. Furthermore, BEAR provides timely outputs and has the very real advantage of enabling dynamic and real-time responses, which are both properties required for regional, national and global AMR preparedness. Realizing the deployment of BEAR in real-world practice will require an understanding of which drug–pathogen combinations it will be suitable for, standardized and transparent methods of eliciting prior belief from infection specialists, access to small but unbiased samples of antimicrobial susceptibility data (e.g. through targeted testing of stored isolates) and continuous review, quality control and updating of BEAR models and algorithms to respond to the dynamic nature of AMR.

## Supplementary Material

dkae230_Supplementary_Data

## Data Availability

The MIMIC-IV version 2.2 dataset is publicly accessible as a credentialled PhysioNet user at https://physionet.org/content/mimiciv/2.2/ once mandated training is completed, and the data use agreement is signed. Additional aggregate-level data can be provided by the authors if requests to do so are in line with legal and ethical data use regulations. Open-source code written for this study is available at https://github.com/amh312/BayesianAMRPrevalence.

## Transparency declarations

A.H. declares personal consulting work for Pfizer outside the submitted work, and a donation from Pfizer to the University of Liverpool for a public and professional engagement project outside the submitted work. W.H. is a member of the Specialist Advisory Committee for GARDP and the Specialty National Co-Lead for Infectious Diseases for the National Institute of Health Research. I.B. holds a senior investigator award with NIHR, an OLS-data-action-accelerator grant, personal fees and other from AstraZeneca outside the submitted work. A.G., A.V., P.L.G., C.B. and D.M.H. have no interests to declare.

## Supplementary data

Figures S1 to S3, Boxes S1 and S2 and Tables S1 to S3 are available as Supplementary data at *JAC* Online.
