# Correction to: Bayesian estimation of the prevalence of antimicrobial resistance: a mathematical modelling study

**DOI:** 10.1093/jac/dkaf494

**Published:** 2026-02-03

This is a correction to: Alex Howard, Peter L Green, Anoop Velluva, Alessandro Gerada, David M Hughes, Charlotte Brookfield, William Hope, Iain Buchan, Bayesian estimation of the prevalence of antimicrobial resistance: a mathematical modelling study, *Journal of Antimicrobial Chemotherapy*, Volume 79, Issue 9, September 2024, Pages 2317–2326, https://doi.org/10.1093/jac/dkae230

The following changes have been made to the published manuscript.

There is an error in the figures used in column 1 of Table 2. The table states that different numbers of tests were used to inform the likelihood. but this was in fact standardised in the final methodology to use 20 specimens for each of the agents, as stated in the methods.

The changes in the Table 2 are as follow:

**Table:** 

		BEAR	BEAR	Observed prevalence	Observed prevalence
Antimicrobial agent	Number of results tested (and simulated)	Mean % AMR prevalence estimation error (SD)	Number of prevalence estimation errors ≥10%	Mean % AMR prevalence estimation error (SD)	Number of prevalence estimation errors ≥10%
Benzylpenicillin	40 (3204) 3224	−2.6 (2.1)	0	0.2 (1.7)	0
Ampicillin	80 (50 396) 50456	0.1 (6.6)	8	−0.3 (8.3)	13
Oxacillin	20 (23 577) 23577	0.4 (7.7)	10	−0.4 (13.1)	21
Ampicillin/sulbactam	60 (49 035) 49075	1.8 (4.2)	0	−0.2 (7.2)	8
Piperacillin/tazobactam	80 (52 747) 52807	4.2 (1.5)	0	−0.1 (1.3)	0
Cefazolin	60 (46 695) 46735	3.5 (4.7)	4	−1.3 (6.1)	6
Cefuroxime	60 (7036) 7076	3.1 (4.1)	1	0.5 (5.2)	1
Ceftriaxone	60 (48 984) 49024	1.9 (4.5)	4	−1.7 (4.9)	1
Ceftazidime	80 (57 708) 57768	2.5 (3.2)	1	0 (4)	1
Cefepime	180 (63 430) 63590	3 (3.2)	1	−0.5 (3.3)	0
Meropenem	180 (63 548) 63708	3.6 (1)	0	0.2 (1.4)	0
Ciprofloxacin	180 (63 571) 63731	1 (4.9)	3	2.2 (6)	6
Levofloxacin	40 (23 673) 23693	−2 (7)	8	−0.6 (11.4)	16
Erythromycin	60 (23 128) 23168	−2.6 (7.5)	10	0.9 (10.7)	17
Clindamycin	40 (21 085) 21105	−0.7 (8.5)	13	−1 (11.2)	23
Tetracycline	40 (17 542) 17562	3.3 (4.1)	5	0.6 (4.6)	1
Vancomycin	20 (11 801) 11801	1.7 (6.9)	4	2.2 (9.8)	20
Rifampicin	20 (8629) 8629	3.7 (2.1)	1	0.4 (3.5)	1
Gentamicin	200 (87 137) 87317	3.2 (2.2)	0	0.1 (3.5)	1
Amikacin	120 (5205) 5305	3.7 (2)	0	−0.1 (2.2)	0
Tobramycin	180 (63 554) 63714	2.8 (2.3)	0	−0.3 (2.9)	0
Nitrofurantoin	140 (47 669) 47789	3.1 (2.3)	1	0 (2.6)	0
Trimethoprim/sulfamethoxazole	220 (79 703) 79903	1.7 (3.5)	0	0.7 (4.9)	0
Total	2160 (919 057) 920757		74		136

In section **Main results**, the second sentence is consequently corrected to read: “Each algorithm simulated 920757 deleted results per cross-validation iteration.” instead of: “Each algorithm used 2160 observed results to simulate 919 057 deleted results per cross-validation iteration.”

