# Supplementary material for: Bayesian estimation of the prevalence of antimicrobial resistance: a mathematical modelling study
Source: J Antimicrob Chemother. 2024 Jul 25;79(9):2317–26. doi: 10.1093/jac/dkae230 (PMC11368424; doi:10.1093/jac/dkae230)
Supplement: dkae230_Supplementary_Data [file dkae230_supplementary_data.docx]

## **Supplementary material**

| Parameters for a beta-distributed prior probability distribution (a continuous distribution that is the conjugate prior for the binomial probability distribution and can only support values between zero and one) were selected informed by literature review – the aim of the search strategy was to discover an AMR prevalence for each drug-pathogen combination from a comparable population to those as the study dataset. Advanced searches of PubMed, Ovid MEDLINE, and Google Scholar literature databases for articles in all languages published between 2008 and 2020 (to cover the timeframe of the study dataset) were performed using a combination of the antimicrobial name, organism name, resistance, and either ‘rate’ or ‘prevalence’. Results were manually searched for studies from the United States initially, then, if necessary, comparable Western nations (e.g. the United Kingdom), then globally. If required, the search was then repeated with no date constraints. Authors then assessed the relevance of the literature to the study setting to produce an appropriately contextualised, moderately informative prior reflecting belief in a very low, low, medium, high, or very high risk of AMR for each drug-pathogen combination (see Figure S1). Authors were blinded to actual population resistance prevalence until after priors had been finalised. Table S1 lists the priors selected for each drug-pathogen combination. |
| --- |

**Box S1**: A description of the process used to select priors for Bayesian estimation of the prevalence of antimicrobial resistance.

**Figure S1**: The cognitive process used to subjectively select one of five moderately informative priors based on a combination of literature review and specialist knowledge. The $\alpha$ and $\beta$ parameters control the shape of the beta prior probability distribution.

| **Antimicrobial agent(s)** |  | **Organism(s)** | **Prior AMR probability belief** |
| --- | --- | --- | --- |
| Benzylpenicillin |  | *Streptococcus pneumoniae* | Very low |
| Benzylpenicillin |  | *Staphylococcus aureus* | Very high |
| Ampicillin |  | *S pneumoniae* | Very low |
| Ampicillin |  | Unknown *Enterococcus* spp*.* | Low |
| Ampicillin |  | *Escherichia coli* | Medium |
| Ampicillin |  | *Proteus mirabilis* | Low |
| Oxacillin |  | *S aureus* | Low |
| Ampicillin-sulbactam |  | *E coli* | Low |
| Ampicillin-sulbactam |  | *Klebsiella pneumoniae* | Low |
| Ampicillin-sulbactam |  | *P mirabilis* | Low |
| Piperacillin-tazobactam |  | *E coli* | Very low |
| Piperacillin-tazobactam |  | *K pneumoniae* | Very low |
| Piperacillin-tazobactam |  | *P mirabilis* | Very low |
| Piperacillin-tazobactam |  | *Pseudomonas aeruginosa* | Very low |
| Cefazolin |  | *E coli* | Low |
| Cefazolin |  | *K pneumoniae* | Low |
| Cefazolin |  | *P mirabilis* | Low |
| Cefuroxime |  | *E coli* | Very low |
| Cefuroxime |  | *K pneumoniae* | Very low |
| Cefuroxime |  | *P mirabilis* | Very low |
| Ceftriaxone |  | *E coli* | Very low |
| Ceftriaxone |  | *K pneumoniae* | Very low |
| Ceftriaxone |  | *P mirabilis* | Very low |
| Ceftazidime |  | *E coli* | Very low |
| Ceftazidime |  | *K pneumoniae* | Very low |
| Ceftazidime |  | *P mirabilis* | Very low |
| Ceftazidime |  | *P aeruginosa* | Very low |
| Cefepime |  | *Citrobacter freundii* | Very low |
| Cefepime |  | *Enterobacter cloacae* | Very low |
| Cefepime |  | *E coli* | Very low |
| Cefepime |  | *K pneumoniae* | Very low |
| Cefepime |  | *Morganella morganii* | Very low |
| Cefepime |  | *P mirabilis* | Very low |
| Cefepime |  | *P aeruginosa* | Very low |
| Cefepime |  | *Serratia marcescens* | Very low |
| Meropenem |  | *C freundii* | Very low |
| Meropenem |  | *E cloacae* | Very low |
| Meropenem |  | *E coli* | Very low |
| Meropenem |  | *K pneumoniae* | Very low |
| Meropenem |  | *M morganii* | Very low |
| Meropenem |  | *P mirabilis* | Very low |
| Meropenem |  | *P aeruginosa* | Very low |
| Meropenem |  | *S marcescens* | Very low |
| Ciprofloxacin |  | *C freundii* | Low |
| Ciprofloxacin |  | *E cloacae* | Low |
| Ciprofloxacin |  | *E coli* | Low |
| Ciprofloxacin |  | *K pneumoniae* | Low |
| Ciprofloxacin |  | *M morganii* | Low |
| Ciprofloxacin |  | *P mirabilis* | Low |
| Ciprofloxacin |  | *P aeruginosa* | Low |
| Ciprofloxacin |  | *S marcescens* | Low |
| Levofloxacin |  | *S pneumoniae* | Very low |
| Levofloxacin |  | *S aureus* | Low |
| Erythromycin |  | *S pneumoniae* | Low |
| Erythromycin |  | Group B *Streptococcus* sp. | Medium |
| Erythromycin |  | *S aureus* | Medium |
| Clindamycin |  | Group B *Streptococcus* sp. | Low |
| Clindamycin |  | *S aureus* | Low |
| Tetracycline |  | *S pneumoniae* | Low |
| Tetracycline |  | *S aureus* | Very low |
| Vancomycin |  | *Enterococcus* spp. | Low |
| Rifampicin |  | *S aureus* | Very low |
| Gentamicin |  | *C freundii* | Very low |
| Gentamicin |  | *Enterobacter cloacae* | Very low |
| Gentamicin |  | *E coli* | Very low |
| Gentamicin |  | *K pneumoniae* | Very low |
| Gentamicin |  | *M morganii* | Very low |
| Gentamicin |  | *P mirabilis* | Very low |
| Gentamicin |  | *P aeruginosa* | Very low |
| Gentamicin |  | *S marcescens* | Very low |
| Gentamicin |  | *S aureus* | Very low |
| Amikacin |  | *E cloacae* | Very low |
| Amikacin |  | *E coli* | Very low |
| Amikacin |  | *K pneumoniae* | Very low |
| Amikacin |  | *P mirabilis* | Very low |
| Amikacin |  | *P aeruginosa* | Very low |
| Tobramycin |  | *C freundii* | Very low |
| Tobramycin |  | *E cloacae* | Very low |
| Tobramycin |  | *E coli* | Very low |
| Tobramycin |  | *K pneumoniae* | Very low |
| Tobramycin |  | *M morganii* | Very low |
| Tobramycin |  | *P mirabilis* | Very low |
| Tobramycin |  | *P aeruginosa* | Very low |
| Tobramycin |  | *S marcescens* | Very low |
| Nitrofurantoin |  | *C freundii* | Very low |
| Nitrofurantoin |  | *E cloacae* | Very low |
| Nitrofurantoin |  | *Enterococcus* spp. | Very low |
| Nitrofurantoin |  | *E coli* | Very low |
| Nitrofurantoin |  | *K pneumoniae* | Very low |
| Nitrofurantoin |  | *S aureus* | Very low |
| Trimethoprim- sulfamethoxazole |  | *C freundii* | Low |
| Trimethoprim- sulfamethoxazole |  | *E cloacae* | Low |
| Trimethoprim- sulfamethoxazole |  | *E coli* | Low |
| Trimethoprim- sulfamethoxazole |  | *K pneumoniae* | Low |
| Trimethoprim- sulfamethoxazole |  | *M morganii* | Low |
| Trimethoprim- sulfamethoxazole |  | *P mirabilis* | Low |
| Trimethoprim- sulfamethoxazole |  | *S marcescens* | Low |
| Trimethoprim- sulfamethoxazole |  | *S aureus* | Very low |
| Trimethoprim- sulfamethoxazole |  | *Stenotrophomonas maltophilia* | Very low |
| Trimethoprim- sulfamethoxazole |  | *S pneumoniae* | Low |

**Table S1**: Authors’ prior belief of AMR prevalence in the Boston-based population for all drug-pathogen combinations assessed.

| Defining $\theta$ as the probability of a resistant result, the prior was chosen to be   \| $P\left( \theta\right) \propto\theta^{\alpha-1} \left( 1-\theta\right)^{\beta-1}$ \| Eq (1) \| \| --- \| --- \|   where $\alpha$ and $\beta$ are shape parameters of the Beta distribution. Defining $x$ as the number of observed resistant results and $N$ as the total number of observed results, the likelihood is given by the binomial distribution   \| $P\left( x \vert N,\theta\right)\propto\theta^{x} ({1-\theta)}^{N-x}$ \| Eq (2) \| \| --- \| --- \|   The posterior can then be shown to be   \| $P\left( \theta\vert x,N \right)\propto\theta^{\alpha+x-1} {(1-\theta)}^{\beta+N-x-1}$ \| Eq (3) \| \| --- \| --- \|   Defining $\bar{x}$ as a sample from the posterior predictive, the posterior predictive can then be shown to be   \| $\mathrm{BetaBin}(\bar{x}\vert\alpha^{'},\beta')$ \| Eq (4) \| \| --- \| --- \|   where $\alpha^{'}= \alpha+x-1$ and $\beta^{'}= \beta+N-x-1$ |
| --- | --- | --- | --- | --- | --- | --- | --- | --- |

**Box S2**: Calculations used to inform estimates


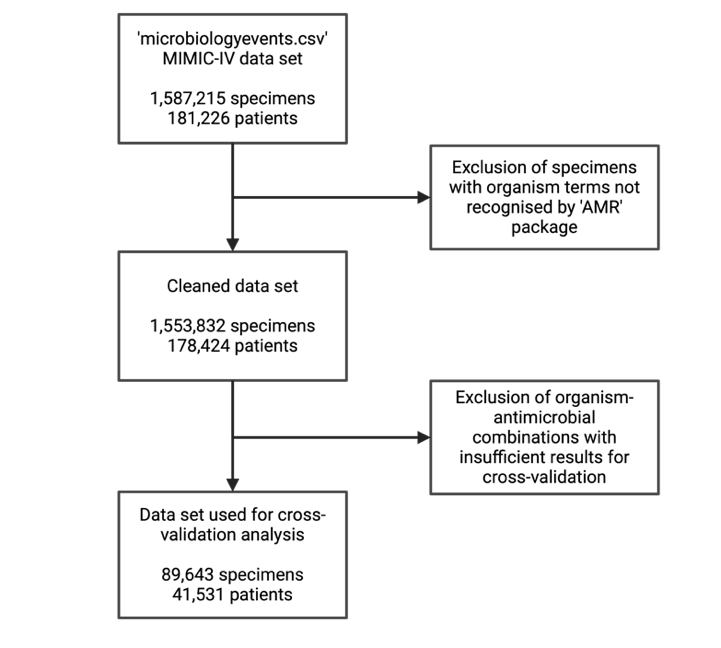


**Figure S2**: Flow chart for the main study analysis

| Antimicrobial agent | Actual AMR prevalence (%) | BEAR mean estimated % AMR prevalence (SD) | Observed prevalence mean estimated % AMR prevalence (SD) |
| --- | --- | --- | --- |
| Benzylpenicillin | 81.1 | 78.6 (2) | 81.4 (1.7) |
| Ampicillin | 43.5 | 43.6 (6.6) | 43.1 (8.3) |
| Oxacillin | 31.8 | 32.3 (7.7) | 31.4 (13.1) |
| Ampicillin-sulbactam | 21.7 | 23.5 (4.2) | 21.5 (7.2) |
| Piperacillin-tazobactam | 3.1 | 7.3 (1.5) | 3 (1.3) |
| Cefazolin | 21 | 24.5 (4.7) | 19.7 (6.1) |
| Cefuroxime | 10.9 | 14 (4.1) | 11.3 (5.2) |
| Ceftriaxone | 12.4 | 14.4 (4.5) | 10.8 (4.9) |
| Ceftazidime | 8.8 | 11.3 (3.2) | 8.8 (4) |
| Cefepime | 8 | 11 (3.2) | 7.4 (3.3) |
| Meropenem | 2.5 | 6.2 (1) | 2.8 (1.4) |
| Ciprofloxacin | 25.8 | 26.8 (4.9) | 27.9 (6) |
| Levofloxacin | 34 | 32 (7) | 33.4 (11.4) |
| Erythromycin | 55.1 | 52.6 (7.5) | 56.1 (10.7) |
| Clindamycin | 36.1 | 35.4 (8.5) | 35.1 (11.2) |
| Tetracycline | 6.4 | 9.7 (4.1) | 7 (4.6) |
| Vancomycin | 27.9 | 29.6 (6.8) | 30.2 (9.8) |
| Rifampicin | 1.6 | 5.3 (2.1) | 2 (3.5) |
| Gentamicin | 7.6 | 10.8 (2.2) | 7.7 (3.5) |
| Amikacin | 3.7 | 7.4 (2) | 3.6 (2.2) |
| Tobramycin | 5.6 | 8.4 (2.3) | 5.3 (2.9) |
| Nitrofurantoin | 7.3 | 10.5 (2.2) | 7.3 (2.6) |
| Trimethoprim-sulfamethoxazole | 19.3 | 21 (3.4) | 20 (4.8) |

**Table S2**: A summary of predicted and actual AMR prevalence across the cross-validation of BEAR and the observed prevalence approach.

|  | **BEAR** | **BEAR** | **Observed prevalence** | **Observed prevalence** |
| --- | --- | --- | --- | --- |
| **Antimicrobial agent** | **Mean AMR prevalence estimation error (SD)** | **Number of estimation errors ≥ 10%** | **Mean AMR prevalence estimation error (SD)** | **Proportion of estimation errors ≥ 10%** |
| **Benzylpenicillin** | -2.5 (1.8) | 0 | -0.1 (2.5) | 0 |
| **Ampicillin** | -0.2 (6.4) | 5 | 0.2 (7.8) | 10 |
| **Oxacillin** | -1.5 (7.4) | 8 | 1.4 (8.3) | 10 |
| **Ampicillin-sulbactam** | -2.2 (4.6) | 2 | -1.3 (9) | 13 |
| **Piperacillin-tazobactam** | 3.2 (1.5) | 0 | 0.2 (2.8) | 0 |
| **Cefazolin** | 3.3 (4.9) | 3 | -0.2 (6.5) | 7 |
| **Cefuroxime** | 1.8 (6.7) | 9 | 0.2 (6.4) | 7 |
| **Ceftriaxone** | 1.5 (4.7) | 2 | -0.6 (5.6) | 4 |
| **Ceftazidime** | 2.2 (3.5) | 2 | 0 (4.1) | 0 |
| **Cefepime** | 2 (3.9) | 2 | -0.1 (4.3) | 1 |
| **Meropenem** | 3.3 (1.1) | 0 | 0.2 (1.4) | 0 |
| **Ciprofloxacin** | -0.3 (4.1) | 0 | -0.6 (7.2) | 9 |
| **Levofloxacin** | -1.7 (7.6) | 11 | 0.7 (10.5) | 14 |
| **Erythromycin** | -0.3 (8.5) | 9 | -0.7 (9.5) | 17 |
| **Clindamycin** | -3.9 (6.9) | 7 | 1.1 (9.8) | 16 |
| **Tetracycline** | 3 (4.6) | 6 | 0.3 (5.5) | 3 |
| **Vancomycin** | -0.2 (5.8) | 4 | -2.8 (8) | 11 |
| **Rifampicin** | 4 (2.3) | 2 | -0.2 (2.5) | 0 |
| **Gentamicin** | 2.3 (2.9) | 0 | 0.1 (3) | 0 |
| **Amikacin** | 3 (1.8) | 1 | 0 (2.4) | 0 |
| **Tobramycin** | 1.6 (3.1) | 1 | 0.2 (3.7) | 0 |
| **Nitrofurantoin** | 1.6 (3.2) | 1 | 0.4 (4.2) | 3 |
| **Trimethoprim-sulfamethoxazole** | 2 (2.9) | 0 | -1.1 (5.2) | 2 |
| **Total** | ·· | 75 | ·· | 127 |

**Table S3**: Results of the sensitivity analysis where all ‘intermediate’ results were assigned to resistant in the MIMIC-IV dataset before running the BEAR and the observed prevalence approach algorithms.


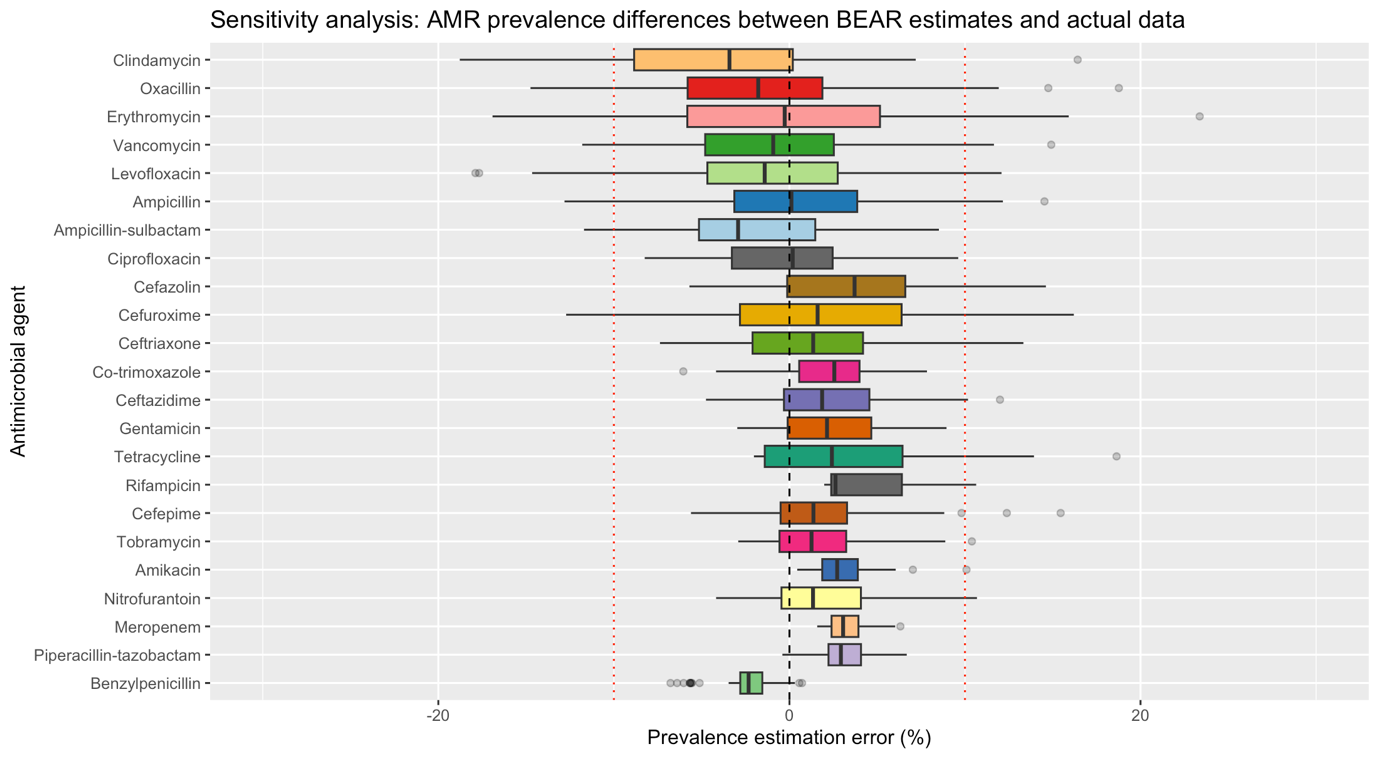


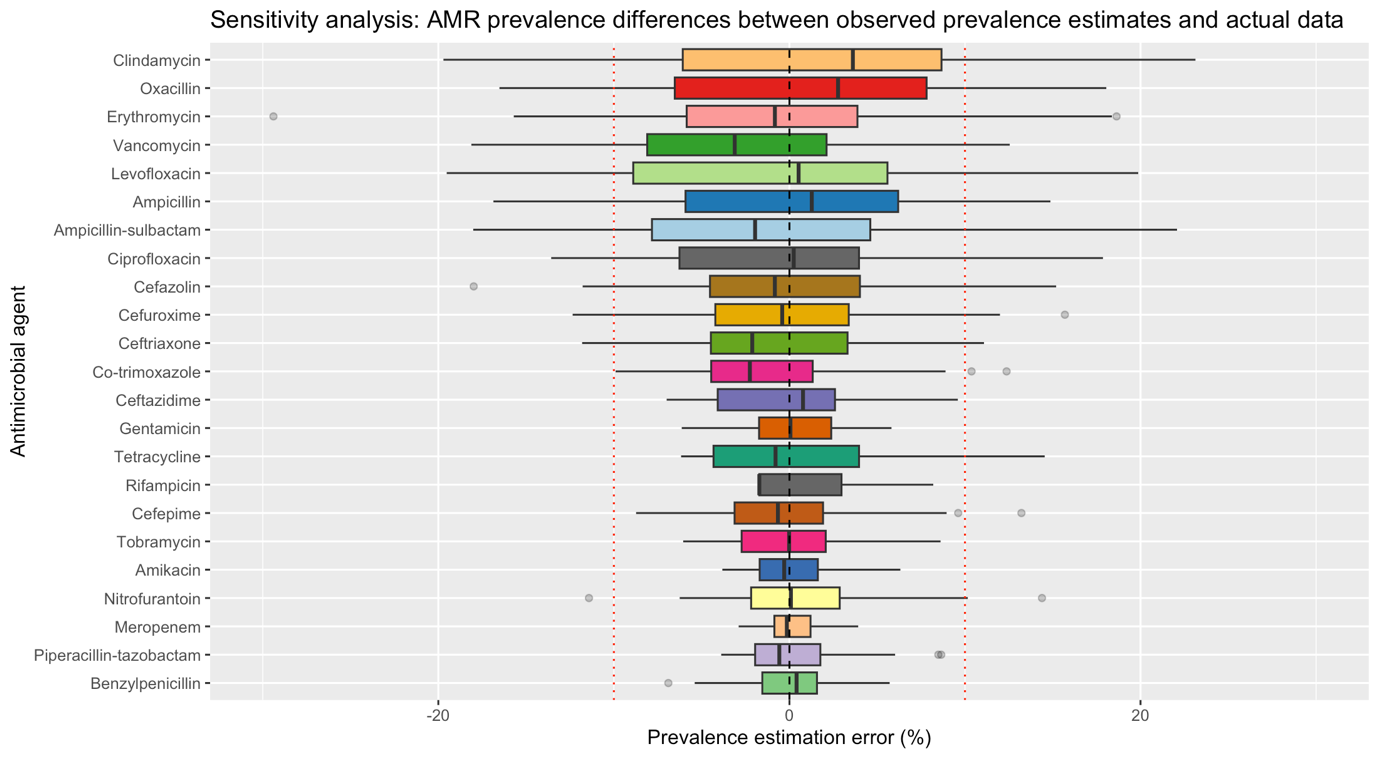


**Figure S3**: Results of the sensitivity analysis where all ‘intermediate’ results were assigned to resistant in the MIMIC-IV dataset before running the BEAR and the observed prevalence approach algorithms.
